# Identification of transcriptional programs using dense vector representations defined by mutual information with GeneVector

**DOI:** 10.1038/s41467-023-39985-2

**Published:** 2023-07-20

**Authors:** Nicholas Ceglia, Zachary Sethna, Samuel S. Freeman, Florian Uhlitz, Viktoria Bojilova, Nicole Rusk, Bharat Burman, Andrew Chow, Sohrab Salehi, Farhia Kabeer, Samuel Aparicio, Benjamin D. Greenbaum, Sohrab P. Shah, Andrew McPherson

**Affiliations:** 1 Computational Oncology, Department of Epidemiology and Biostatistics, Memorial Sloan Kettering Cancer Center, New York, NY USA; 2 Immuno-Oncology Service, Human Oncology and Pathogenesis Program, Memorial Sloan Kettering Cancer Center, New York, NY USA; 3 Hepatopancreatobiliary Service, Department of Surgery, Memorial Sloan Kettering Cancer Center, New York, NY USA; 4 Department of Medicine, Memorial Sloan Kettering Cancer Center, New York, NY USA; 5 Department of Medicine, Thoracic Oncology Service, Memorial Sloan Kettering Cancer Center, New York, NY USA; 6 Department of Molecular Oncology, British Columbia Cancer Research Centre, Vancouver, British Columbia, Canada; 7 Department of Pathology and Laboratory Medicine, University of British Columbia, Vancouver, British Columbia, Canada; 8 Physiology, Biophysics & Systems Biology, Weill Cornell Medicine, Weill Cornell Medical College, New York, NY USA

**Keywords:** Machine learning, Software, Cancer genomics, Tumour heterogeneity, Transcriptomics

## Abstract

Deciphering individual cell phenotypes from cell-specific transcriptional processes requires high dimensional single cell RNA sequencing. However, current dimensionality reduction methods aggregate sparse gene information across cells, without directly measuring the relationships that exist between genes. By performing dimensionality reduction with respect to gene co-expression, low-dimensional features can model these gene-specific relationships and leverage shared signal to overcome sparsity. We describe GeneVector, a scalable framework for dimensionality reduction implemented as a vector space model using mutual information between gene expression. Unlike other methods, including principal component analysis and variational autoencoders, GeneVector uses latent space arithmetic in a lower dimensional gene embedding to identify transcriptional programs and classify cell types. In this work, we show in four single cell RNA-seq datasets that GeneVector was able to capture phenotype-specific pathways, perform batch effect correction, interactively annotate cell types, and identify pathway variation with treatment over time.

## Abstract (web summary)

In single-cell RNA-seq analyses, it would be critical to measure the relationships between genes. Here, the authors develop a framework for single-cell dimensionality reduction that incorporates gene-specific relationships - GeneVector -, and use it for tasks such as annotating cell types and analysing pathway variation after treatment.

## Introduction

Maintenance of cell state and execution of cellular function are based on coordinated activity within networks of related genes. To approximate these connections, transcriptomic studies have conceptually organized the transcriptome into sets of co-regulated genes, termed gene programs^1^ or metagenes^2^. The first intuitive step to identify such co-regulated genes is the reduction of dimensionality for sparse expression measurements: high dimensional gene expression data is compressed into a minimal set of explanatory features that highlight similarities in cellular function. However, to map existing biological knowledge to each cell, the derived features must be interpretable at the gene level.

To find similarities in lower dimensions, biology can borrow from the field of natural language processing (NLP). NLP commonly uses dimensionality reduction to identify word associations within a body of text^3,4^. To find contextually similar words, NLP methods make use of vector space models to represent similarities in a lower dimensional space. Similar methodology has been applied to bulk RNA-seq expression for finding co-expression patterns^5^. Inspired by such work, we developed a tool that generates gene vectors based on single cell RNA (scRNA)-seq expression data. While current methods reduce dimensionality with respect to sparse expression across each cell, our tool produces a lower dimensional embedding with respect to each gene. The vectors derived from GeneVector provide a framework for identifying metagenes within a gene co-expression graph and relating these metagenes back to each cell using latent space arithmetic.

The most pervasive method for identifying the sources of variation in scRNA-seq studies is principal component analysis (PCA)^6–8^. The relationship of principal components to gene expression is linear, allowing lower dimensional structure to be directly related to variation in expression. A PCA embedding is an ideal input for building a nearest neighbor graph for unsupervised clustering algorithms^9^ and visualization methods including t-SNE^10^ and UMAP^11^. However, the assumption of a continuous multivariate gaussian distribution creates distortion in modeling read counts generated by a true distribution that is over-dispersed, possibly zero-inflated^12^, with positive support and mean close to zero^2^. Despite such issues, gene programs generated from PCA loadings have been used to generate metagenes that explain each principal component^13^. While these loadings highlight sets of genes that explain each orthogonal axis of variation, pathways and cell type signatures can be conflated within a single axis.

In addition to PCA, more sophisticated methods have been developed to better handle the specific challenges of scRNA data. The single cell variational inference (scVI) framework^14^ generates an embedding using non-linear autoencoders that can be used in a range of analyses including normalization, batch correction, gene-dropout correction, and visualization. While scVI embeddings show improved performance over traditional PCA-based analysis in these tasks, they have a non-linear relationship to the original count matrix that may distort the link between structure in the generated embedding and potentially identifiable gene programs^2^. A subsequent method uses a linearly decoded variational autoencoder (LDVAE), which combines a variational autoencoder with a factor model of negative binomial distributed read counts to learn an interpretable linear embedding of cell expression profiles^2^. However, the relationship between gene expression and cell representation is still tied to correlated variation across cells, which may confound co-varying pathway and phenotypic signatures.

Recognizing the importance of modeling the non-linearity of gene expression and the complexity of statistical dependencies between genes, several methods have adopted information theoretical approaches. Many of these methods use mutual information (MI), an information theoretic measure of the statistical dependence between two variables. ARACNE^15^ uses MI to prune independent and indirectly interacting genes during construction of a gene regulatory network from microarray expression data. PIDC^16^ identifies regulatory relationships using partial information decomposition (PID), a measure of the dependence between triples of variables. The authors apply PIDC to relatively high-depth single-cell qPCR datasets and restrict their analysis to on the order of hundreds of genes. More recently, IQCELL^17^ uses MAGIC^18^ to impute missing gene expression, builds a GRN from pairwise MI between genes, and applies a series of filters to produce a GRN composed of only functional relationships. The authors use IQCELL to identify known causal gene interactions in scRNA-seq data from mouse T-cell and red blood cell development experiments. Because of the success of these methods, we hypothesized that MI could be combined with vector space models to produce a meaningful low dimensional representation of genes from scRNA data.

**Fig. 1:**
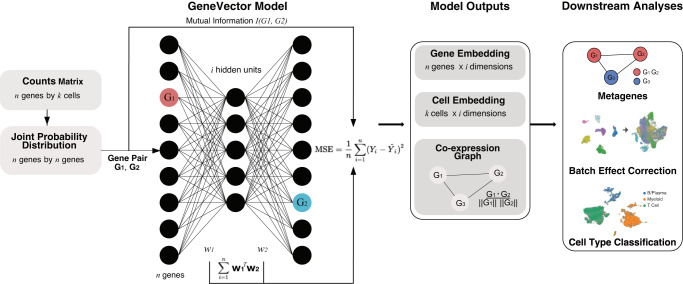
GeneVector Framework. Overview of GeneVector framework starting from single cell read counts. Mutual information is computed on the joint probability distribution of read counts for each gene pair. Each pair is used to train a single layer neural network where the MSE loss is evaluated from the model output (**w**_1_^T^**w**_2_) with the mutual information between genes. From the resulting weight matrix, a gene embedding, cell embedding, and co-expression similarity graph are constructed. Using vector space arithmetic, downstream analyses include identification of cell-specific metagenes, batch effect correction, and cell type classification.

In this work, we present GeneVector (Fig. 1) as a framework for generating low dimensional embeddings constructed from the mutual information between genes. GeneVector summarizes co-expression of genes as mutual information between the probability distribution of read counts across cells. We showcase GeneVector on four scRNA datasets produced from a diverse set of experiments: peripheral blood mononuclear cells (PBMCs) subjected to interferon beta stimulation^19^, the Tumor Immune Cell Atlas (TICA)^20^, treatment naive multi-site samples from High Grade Serous Ovarian Cancers (HGSOC)^21^ and a time series of cisplatin treatment in patient-derived xenografts (PDX) of triple negative breast cancer (TNBC)^22^. We first confirm GeneVector’s ability to identify putatively co-regulated gene pairs from sparse single cell expression measurements using the TICA and PBMC datasets. We demonstrate that latent space arithmetic can be used to accurately label cell types in the TICA dataset and validate our cell type predictions against published annotations. Next, we show that GeneVector can identify metagenes corresponding to cell-specific transcriptional processes in PBMCs, including interferon activated gene expression (ISG). We use vector space arithmetic to directly map metagenes to site specific changes in primary and metastatic sites in the HGSOC dataset, capturing changes in MHC class I expression and epithelial-mesenchymal transition (EMT). Finally, we show GeneVector can identify cisplatin treatment dependent transcriptional programs related to TGF-beta in TNBC PDXs.

## Results

### Defining the GeneVector framework

We trained a single layer neural network over all gene pairs to generate low dimensional gene embeddings and identify metagenes from a co-expression similarity graph. The input weights (**w**_1_) and output weights (**w**_2_) are updated with adaptive gradient descent (ADADELTA)^23^. Gene co-expression relationships are defined using mutual information (Methods**:** Mutual Information) computed from a joint probability distribution of expression counts. Training loss is evaluated as the mean squared error of mutual information with the model output, defined as **w**_1_^T^**w**_2_. The final latent space is a matrix defined as a series of vectors for each gene.

Gene vectors produced by the framework are useful for several fundamental gene expression analyses. Gene vectors weighted by expression in each cell are combined to generate the cell embedding analysis of cell populations and their relationships to experimental covariates. The cell embedding can be batch corrected by using vector arithmetic to identify vectors that represent batch effects and then shift cells in the opposite direction (Methods**:** Batch correction). A co-expression graph is constructed in which each node is defined as a gene and each edge is weighted by cosine similarity. After generating the co-expression graph, we use Leiden clustering^9^ to identify metagene clusters. Further downstream analysis of the cell embedding includes phenotype assignment based on sets of marker genes and computation of the distances between cells and metagenes to highlight changes related to experimental covariates (Fig. 1).

To perform cell type assignment, a set of known marker genes is used to generate a representative vector for each cell type, where each gene vector is weighted by the normalized and log-transformed gene expression. The cosine similarity of each possible phenotype is computed between the cell vector and the marker gene vector. SoftMax is applied to cosine distances to obtain a pseudo-probability over each phenotype (Methods: Cell type assignment). Discrete labels can be assigned to cells by selecting the phenotype corresponding to the maximum pseudo-probability. More generally, gene vectors can be composed together to describe interesting gene expression features. Cell or gene vectors can then be compared against these feature vectors to evaluate the relevance of that feature to a given cell or gene (Methods: Generation of Predictive Genes).

### Robust inference of gene co-regulation with GeneVector

**Fig. 2:**
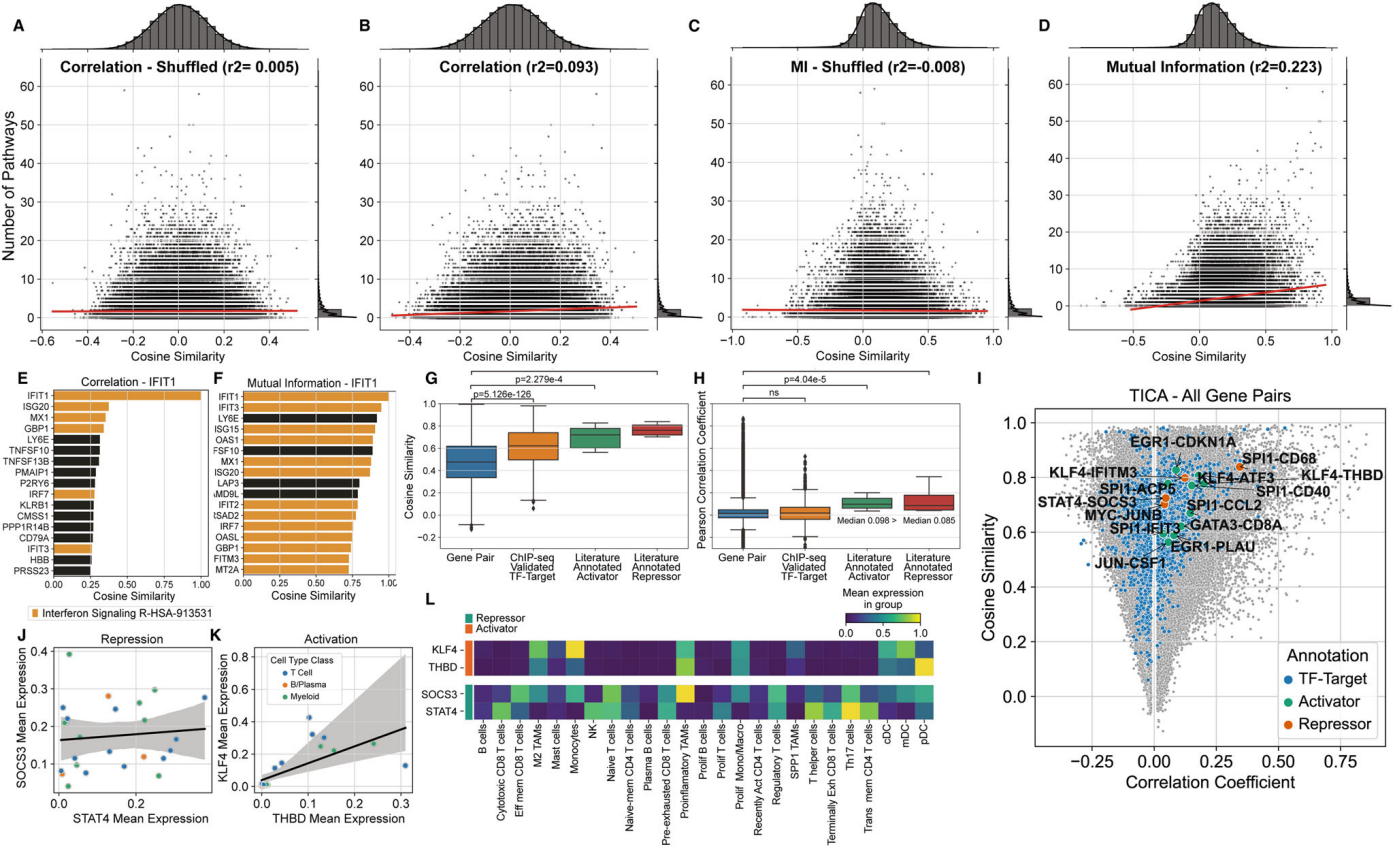
Comparison of Results using Mutual Information. **A**–**D** Pathway co-membership vs. cosine similarity between gene vectors for all gene pairs in PBMCs. Each point represents one gene pair, and plots show the number of pathways (combined Reactome and MSigDB cell type signatures [C8]) that contain both genes (y-axis) and the cosine distance between the two genes (x-axis). The results show both correlation **A**, **B** and MI (Mutual Information) **C**, **D** based GeneVector. In addition to a standard set of results **B**, **D**, a baseline relationship between pathway co-membership and cosine similarity is established by performing an identical analysis over randomly shuffled gene **A**, **C**. **E** Top 16 most similar genes by cosine similarity to IFIT1 using correlation coefficient. Genes in the interferon signaling pathway are colored orange. **F** Top 16 most similar genes to IFIT1 after training GeneVector using mutual information shows a higher number of interferon signaling pathway genes. **G**, **H** Cosine similarity and Pearson correlation coefficient for un-annotated gene pairs (*n* = 314090), ChIP-Seq annotated TF-targets pairs (*n* = 1275), and literature annotated activator (*n* = 26) or repressor (*n* = 26) TF-target pairs. The center of the box plot is denoted by the median, a horizontal line dividing the box into two equal halves. The bounds of the box are defined by the lower quartile (25th percentile) and the upper quartile (75th percentile). The whiskers extend from the box and represent the data points that fall within 1.5 times the interquartile range (IQR) from the lower and upper quartiles. Any data point outside this range is considered an outlier and plotted individually. Significance assessed using Mann-Whitney-Wilcoxon two-sided test. **I** Cosine similarity versus correlation coefficient for gene pairs in the TICA (Tumor Immune Cell Atlas) dataset with TF-target gene pairs highlighted (blue) and colored by activator/repressor status (green/orange respectively). **J**, **K** Linear regression of mean log-normalized expression per cell type±95% confidence interval for repressor TF-target pair *SOCS3*-*STAT4* and activator TF-target pair *KLF-THBD*, respectively. **L** Mean log-normalized expression for *SOCS3-STAT4* and *KLF4-THBD* across annotated cell types. Source data provided as a Source Data file.

Our model relies on the advantages of mutual information to define relationships between genes, as opposed to other distance metrics. To evaluate how MI contributes to the observed performance of the model, we first validated that the vectors inferred by GeneVector capture semantic qualities of genes including pathway memberships and regulatory relationships. Specifically, we assessed the extent to which pairs of genes within the same pathway, or expressed within the same cell types, produced similar vector representations in the PBMC dataset. As a ground truth we computed, for each gene pair, the number of combined pathways from Reactome^24^ and MSigDB^25,26^ cell type signatures (C8) for which both genes were members. In addition to training GeneVector using raw read counts with an MI target, we trained GeneVector using normalized and log-transformed read counts on Pearson correlation coefficient to evaluate the relative benefit of MI on the accuracy of the model output. For both the correlation and MI based models, cosine similarities between gene vectors showed a stronger relationship with the number of shared pathways and cell type signatures than randomly shuffled gene pairs (Fig. 2A, C). Comparing the MI model and the correlation model directly, the MI model produced a much stronger relationship than the correlation based model (r^2^ = 0.233 vs. r^2^ = 0.093, Fig. 2B, D). To provide a pathway specific example, we found that the most similar genes by cosine similarity to *IFIT1* (a known interferon stimulated gene) using the correlation objective were less coherent in terms of ISG pathway membership signal (6 of 16 genes were in the Interferon Signaling Reactome pathway R-HSA-913531) (Fig. 2E) than with mutual information (12 of 16 genes) (Fig. 2F). The greater proportion of interferon stimulated pathway genes with high similarity to *IFIT1* using a mutual information objective function (Fig. 2F vs. 2E) is consistent with the improved correlation between pathway co-membership and cosine similarity over the Pearson correlation coefficient objective (Fig. 2D vs. 2FB).

Next, we sought to understand whether GeneVector was able to capture relationships between genes with known interactions such as transcription factors (TF) and their targets. GeneVector cosine similarities of TF-target pairs annotated based on ChIP-Seq data^27^ were significantly increased relative to un-annotated gene pairs in the TICA dataset (Fig. 2G). We also considered literature annotated activator-target and repressor-target TF-target gene pairs^28^. As expected, activator-target pairs showed increased cosine similarity above unannotated pairs. Importantly, repressor-target pairs showed an equally strong increase in cosine similarity above unannotated pairs highlighting GeneVector’s ability to identify a diversity of statistical dependencies between co-regulated genes. By comparison, Pearson correlation coefficients of ChIP-seq annotated TF-target pairs were not significantly different from unannotated gene pairs (Fig. 2H). Both activator-target and repressor-target pairs were significantly different from unannotated pairs, though correlation was on-average positive for both repressor-target and activator-target pairs (Fig. 2H). In fact, while many repressor-target pairs had high cosine similarity indicative of a meaningful regulatory relationship, their correlation coefficients, computed with normalized and log-transformed counts, were always positive (Fig. 2I). For example, *SOCS3*-*STAT4* exhibited the lowest correlation of annotated repressor-target pairs (*r*^2^ = 0.017) and aggregating normalized and log-transformed expression across cell types showed an absence of any relationship between these genes (Fig. 2J). In contrast, analysis of activator-target pair *KLF4*-*THBD* revealed a positive correlation driven by co-expression in myeloid cells and T cells (Fig. 2K). This relationship is further evidenced when looking at the expression in more detailed cell type annotations (Fig. 2L). Identification of mutually exclusive expression is a theoretical benefit of correlation-based similarity measures, however, the sparsity of scRNA likely results in positive or low correlation even for known examples of mutual exclusivity. In summary, the vector space produced by GeneVector successfully recovers the latent similarities between functionally related genes, including negative regulators and their targets, overcoming the sparsity of scRNA data that confounds simpler approaches.

### Fast and accurate cell type classification using GeneVector

**Fig. 3:**
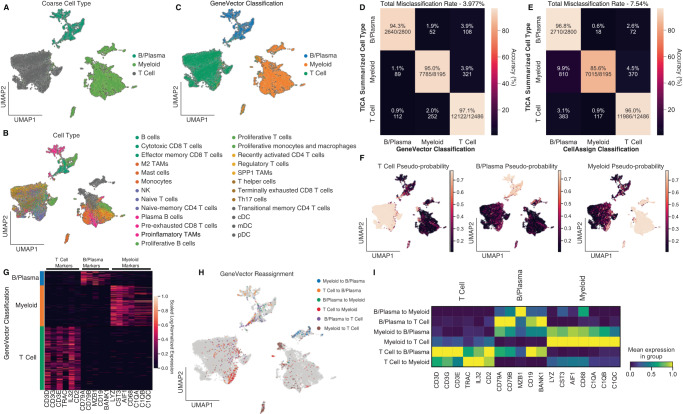
GeneVector Accurately Classifies Cells in TICA Cell Atlas. **A** Cells annotated by cell type summarized from the original TICA provided annotations. **B** Original annotations provided in TICA (Tumor Immune Cell Atlas). **C** GeneVector classification results for each cell type. **D** Confusion matrix comparing GeneVector classification with summarized cell types. **E** Confusion matrix using the same gene markers with CellAssign shows decreased performance in myeloid cells. **F** Pseudo-probability values for each summarized cell type. **G** Marker gene log-normalized expression over all cells grouped by GeneVector classification. **H** Original annotations reassigned by GeneVector. **I** Marker gene mean log-normalized expression for cell type reassignments highlights misclassifications in B/Plasma cells and doublet transcriptional signatures in T and myeloid cells. Source data provided as a Source Data file.

Comparative analysis of gene expression programs across large cohorts of patients can potentially identify transcriptional patterns in common cell types shared between many cancers. However, classification of cell types using methods such as CellAssign^29^ are computationally expensive. Furthermore, the large number of covariates in these datasets makes disentangling patient-specific signals from disease and therapy difficult. GeneVector provides a fast and accurate method of cell type classification. We perform cell type classification on a subset of 23,764 cells from the Tumor Immune Cell Atlas (TICA) composed of 181 patients and 18 cancer types^20^. The dataset was subset to 2000 highly variable genes and the unnormalized read counts were used train GeneVector. Cell vectors were generated by weighting each gene vector by the normalized and log-transformed expression per cell. Cell types were summarized into three main immune cell types: T cells, B/Plasma, and Myeloid cells (Fig. 3A) from the original annotations (Fig. 3B). We selected a set of gene markers for each cell type (T cells: *CD3D*, *CD3G*, *CD3E*, *TRAC*, *IL32*, *CD2*; B cells: *CD79A*, *CD79B*, *MZB1*, *CD19*, *BANK1*; and Myeloid cells: *LYZ*, *CST3*, *AIF1*, *CD68*, *C1QA*, *C1QB*, *C1QC*) based on signatures obtained from CellTypist^30^. For each phenotype and each cell, we computed the cosine distance to the log-normalized expression weighted average of the marker gene vectors. The pseudo-probabilities for the three cell types are generated by applying a SoftMax function to the set of cosine distances. The maximum pseudo-probability is used to classify each cell into T cell, B/Plasma, or Myeloid (Fig. 3C).

To assess performance, we computed accuracy against the coarse labels in a confusion matrix as the percentage of correctly classified cells over the total number cells for each summarized cell type. We found 97.1% of T cells, 95% of myeloid cells, and 94.3% of B/Plasma cells were correctly classified with respect to the original annotations (Fig. 3D). Additionally, we classified cells using the same marker genes with CellAssign and found significantly decreased performance in the classification of myeloid cells (85.6%) (Fig. 3E). Overall, the percentage of cells misclassified using GeneVector (3.977%) showed improvement over CellAssign (7.54%). Using the pseudo-probabilities, GeneVector can highlight cells that share gene signatures including plasmacytoid dendritic cells (pDCs), where cell type definition is difficult^31^ (Fig. 3F). For each cell type, we validated that the classified cells are indeed expressing the supplied markers by showing the normalized and log-transformed expression for each marker grouped by classified cell type (Fig. 3G). For those cells GeneVector reassigned from the original annotations (Fig. 3H), we examined the mean normalized and log-transformed expression per marker gene and found that many of these cells appear misclassified in original annotations (Fig. 3I). Cells originally annotated as T cells that were reassigned as B/Plasma by GeneVector show high expression for only B/Plasma markers (*CD19*, *BANK1*, *CD79A*, and *CD79B*). Additionally, there is evidence that many of these reassigned cells may be doublets. B/Plasma cells reassigned to T or myeloid cells show simultaneous expression of both gene markers. While any computational cell type classification cannot be considered ground truth, cell type assignment with GeneVector is an improvement over CellAssign and demonstrates sensitivity to cells that express overlapping cell type transcriptional signatures.

Next, we benchmarked GeneVector’s cell type assignment performance with respect to four input types: raw read counts, log-transformed raw counts, normalized total counts per cell, and normalized total counts per cell with log-transformation. We evaluated each approach on the original TICA dataset, in addition to a series of datasets for which we artificially generated a library size batch effect by down sampling reads in subsets of cells (Supplementary Fig. 1A–E). Performance was evaluated by calculating cell type assignment accuracy and by ranking gene pairs by cosine similarity for annotated co-expressed and mutually exclusive gene pairs using CellTypist (Methods: Coexpressed and Mutually Exclusive Markers)^30^. Evaluating gene pair rankings on repeat trained models of the full TICA dataset, we find that all four preprocessing types show a similar ranking of co-expressed gene pairs (Supplementary Fig. 1A, left). Interestingly, raw counts produce significantly lower rankings for mutually exclusive gene pairs (Supplementary Fig. 1A, right). When introducing an artificial batch effect, we find that raw counts generate significantly lower rankings for mutually exclusive marker pairs (Supplementary Fig. 2B). Next, we compared the cell type prediction accuracy between normalization procedures using the same gene markers used to perform cell type prediction in the main results section on both the full and subset datasets with artificial batch effect. Raw counts significantly outperformed all other preprocessing inputs by a large margin, with both normalized and log-normalized showing very poor performance (Supplementary Fig. 2C, D). Our results suggest that normalization increases association between pairs of genes with mutually exclusive expression, resulting in negative downstream performance of cell type prediction. Cell typing appears to be sensitive to mutual exclusivity of cell type markers, and normalization produces slight increases in cosine similarity between gene vectors of mutually exclusive genes resulting in poor cell typing accuracy.

After learning the gene embedding, GeneVector allows rapid testing of different marker genes and phenotypes in exploratory analysis settings. Increased performance in classification is important given the large variation of markers used to define the same phenotypes across different studies. Cell type prediction can be recomputed interactively within a Jupyter notebook within twenty seconds for even large datasets on most machines (Supplementary Fig. 2). An additional advantage of having a probability is the ability to map genes from known pathways to a continuous value in each cell. In both phenotype and pathway, demonstration of continuous gradients across cells provides a measure of change and activation that cannot be seen from unsupervised clustering.

### Comparison of Methods in Identification of Interferon Metagenes in 10k Human PBMCs

**Fig. 4:**
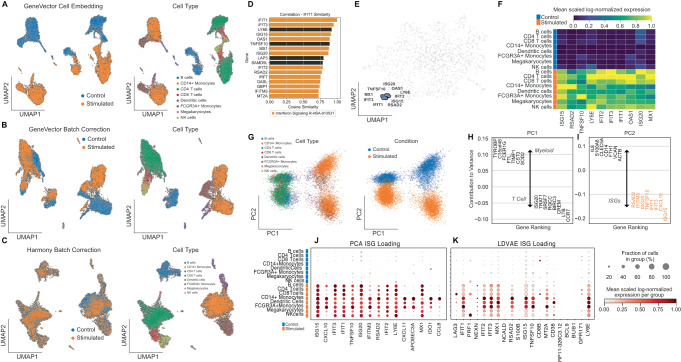
Comparing Methods with Interferon Beta Stimulated 10 K PBMC. **A** Uncorrected GeneVector UMAPs showing stimulated condition (left) and cell type annotation (right) on 10k PBMCs with control and interferon beta stimulated cells. **B** GeneVector batch corrected UMAPs showing stimulated condition (left) and cell type annotation (right) indicated stronger interferon-beta stimulated response in myeloid cells. **C** Harmony batch correction applied to normalized expression eliminates all variation related to interferon beta stimulation. **D** Most similar genes by cosine similarity to *IFIT1* includes genes found in Reactome interferon related pathways. **E** Gene embedding UMAP highlighting an ISG (interferon stimulated genes) metagene that includes genes most similar to *IFIT1*. **F** Scaled log-normalized expression of ISG (interferon stimulated gene) metagene shows increased expression across stimulated cells without cell type specific effects. **G** PCA embedding colored by cell type (left) and stimulation (right). **H** Top genes by contribution to variance indicates PC1 defines cell type. **I** Top genes by contribution to PC2 defined by ISG stimulation colored by scaled normalized, log-transformed gene expression scaled by variable. **J**, **K** Scaled normalized, log-transformed expression scaled by variable of ISG related loadings from PCA (left) and LDVAE (right) includes cell type specific markers intermixed with ISGs. Source data provided as a Source Data file.

To identify cell-specific metagenes related to interferon beta stimulation and compare with transcriptional programs identified by PCA and LDVAE loadings, we trained GeneVector using peripheral blood mononuclear cells (PBMCs) scRNA-seq data from 6855 quality control filtered cells composed of an interferon beta stimulated sample and a control sample^19^. The raw count matrix was subset to 1000 highly variable genes using the Seurat V3 method^32^ as implemented in Scanpy^7^. We used previously annotated cell types generated from unsupervised clustering as ground truth labels^33^. The cell embedding used for batch correction was generated by weighting gene vectors by the normalized, log-transformed expression in each cell. A comparison of the uncorrected UMAP embedding (Fig. 4A) and subsequent GeneVector-based batch correction (Fig. 4B) demonstrates correction in the alignment of cell types between the two conditions. However, in contrast to batch correction using Harmony^34^ (Fig. 4C), not all variation is lost between the interferon beta stimulated and control cells. Specifically, GeneVector does not align myeloid cell types, suggesting a larger effect of the interferon beta stimulation treatment in these cells. Finally, we explored the impact of cell type composition on batch correction performance and found that CD14+ Monocytes had the largest batch silhouette coefficient, indicating that stimulated and control Monocytes differed the most across cell types (Supplementary Fig. 6G).

As a method of both validation and exploration, GeneVector provides the ability to query similarity in genes. For a given target gene, a list of the closest genes sorted by cosine similarity can be generated. This is useful in both validating known markers and identifying the function of unfamiliar genes by context. The genes most similar to IFIT1 (Fig. 4D) include a large proportion of genes found in the Reactome pathway Interferon Signaling (R-I-913531) (Gillespie et al. 2022). After clustering gene vectors, we identify a single metagene that includes these genes (*IFIT1*, *IFIT2*, *IFIT3*, *ISG15*, *ISG20*, *TFGS10*, *RSAD2*, *LYSE*, *OAS1*, and *MX1*). The ISG metagene can be visualized on a UMAP generated from the gene embedding, like the familiar cell-based visualizations common in scRNA-seq studies (Fig. 4E). The mean and scaled log-normalized expression of each gene identified in the ISG metagene is significantly higher in interferon beta stimulated cells over control cells (Fig. 4F). Importantly, the increased expression is found in each cell type, indicating a global relation to treatment.

To compare the ISG metagene with results generated from PCA loadings, we performed PCA on the normalized and log-transformed gene-by-cell expression matrix (Fig. 4G) and colored the embedding by cell type and treatment. After computing the PCA loadings using Scanpy^7^, we identified the top genes by contribution score to variation in the first and second principal components. The first principal component (PC1) explains variation related to cell type and the differences between myeloid (*TYROBP*, *FCER1G*, *FTL*, *CST3*) and T cells *(LTB*, *CCR7*) (Fig. 4H). The second principal highlights the variation related to interferon beta stimulation and includes the genes found in the ISG metagene generated by GeneVector (Fig. 4I). However, the increased effect of interferon stimulation in myeloid cells, conflates myeloid specific ISGs with the interferon signature. One such gene is *CXCL10*, which shows cell type specificity to myeloid cells (Fig. 4J) and is not found in the interferon signaling Reactome pathways. Additionally, *IFITM3* shows increased expression only in myeloid cells within these PBMCs. In contrast, GeneVector produces a metagene that groups myeloid specific genes into a single metagene including *CXCL10* and *IFITM3*. A full list of metagenes produced by GeneVector is presented in Supplementary Fig. 3. Among these metagenes, we identify transcriptional programs specific to each cell type and treatment condition, including those found in the least represented cell type Megakaryocytes (132 of 14,038 cells).

To compare the GeneVector ISG metagene with LDVAE, we trained an LDVAE model using 10 latent dimensions for 250 epochs with control and stimulated batch labels in the SCVI framework on the unnormalized read counts. In contrast to the specificity of the GeneVector ISG metagene that includes only interferon stimulated genes, the nearest LDVAE loading mixes interferon-related genes with markers of T cell activation (*PRF1*) and T cell dysfunction (*LAG3*) (Fig. 4K). With respect to PCA and LDVAE loadings, GeneVector identified an ISG metagene that is not confounded by cell type and includes only interferon pathway related genes.

### Metagenes changes between primary and metastatic site in HGSOC

Studies with scRNA-seq data sampled from multiple tumor sites in the same patient provide a wide picture of cancer progression and spread. As these datasets grow larger and more complex, understanding the transcriptional changes that occur from primary to metastatic sites can help identify mechanisms that aid in the process of the invasion-metastasis cascade. GeneVector provides a framework for asking such questions in the form of latent space arithmetic. By defining the difference between two sites as a vector, where the direction defines transcriptional change, we identify metagenes associated with expression loss and gain between primary and metastasis sites from six patients in the Memorial Sloan Kettering Cancer Center SPECTRUM cohort of patients with high-grade serous ovarian cancer (HGSOC)^21^.

**Fig. 5:**
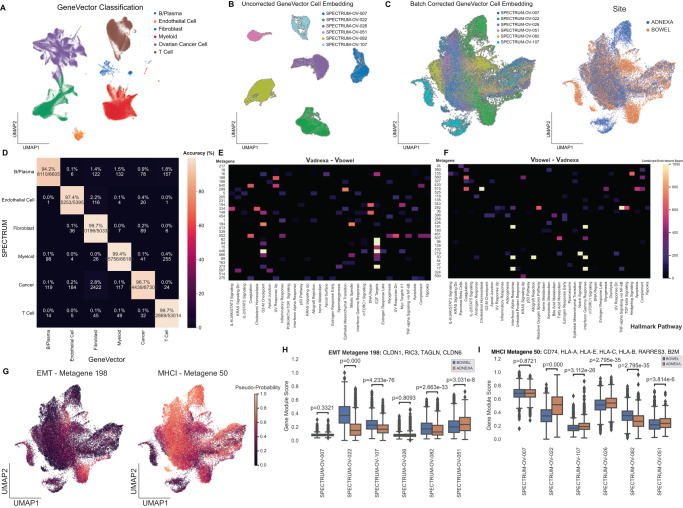
Metagenes Associated with Directional Difference in HGSOC Cancer Cells from Adnexa to Bowel. **A** UMAP of HGSOC cells with classified by GeneVector. **B** Uncorrected UMAP of cancer cells from patients with adnexa and bowel samples. **C** GeneVector batch corrected UMAP with patient labels on site labels on batch corrected UMAP. **D** Confusion matrix of accuracy comparing SPECTRUM annotated cell types with GeneVector classification. **E** Hallmark pathway enrichment for top 30 metagenes by cosine similarity to **V**_adnexa_ – **V**_bowel_. **F** Hallmark pathway enrichment for top 30 metagenes by cosine similarity to **V**_bowel_ – **V**_adnexa_. **G** Pseudo-probabilities for metagenes associated with up-regulation in bowel to adnexa (Epithelial-to-Mesenchymal Transition) and down-regulation (Major Histocompatibility Class I). **H** EMT (Epithelial-Mesenchymal Transition) metagene significantly up regulated in four of six patients by gene module score. **I** MHC Class I (MHCI) metagene significantly downregulated in the metastatic site (bowel) in three of six patients by gene module score. Source data provided as a Source Data file. The center of the box plot is denoted by the median, a horizontal line dividing the box into two equal halves. The bounds of the box are defined by the lower quartile (25th percentile) and the upper quartile (75th percentile). The whiskers extend from the box and represent the data points that fall within 1.5 times the interquartile range (IQR) from the lower and upper quartiles. Any data point outside this range is considered an outlier and plotted separately. Significance assessed using Mann-Whitney-Wilcoxon two-sided test.

A set of 270,833 cells quality control filtered cells from adnexa (primary) and bowel (metastasis) samples were processed with GeneVector (Fig. 5A). The unnormalized counts were subset to 2000 highly variable genes were used as input to GeneVector and cells were classified to one of six cell types using gene markers curated for HGSOC and two markers for cancer cells (*EPCAM* and *CD24*)^21^. We performed cell type classification (Methods: Cell Type Assignment) and compared GeneVector accuracy to the original annotations generated from CellAssign for each cell type in a confusion matrix (Fig. 5B). Accuracy reached 99.7% in three cell types with a minimum classification rate of 94.1%. In cells where GeneVector annotated differently than previous annotations, there is evidence from the differentially expressed genes that these cells may have been initially mislabeled. GeneVector reassigned a subset of cancer cells to fibroblast and the differentially expressed genes between these cells and the cells annotated as cancer by GeneVector highlighted fibroblast cell type markers including *COL1A1* (Supplementary Fig. 4A). In B/Plasma cells reassigned as T cells, the differentially expressed genes highlight B cell receptor genes *IGK* and *IGLC2* (Supplementary Fig. 4B). Finally, in B/Plasma cells classified as myeloid, the top differentially expressed genes include known canonical markers for macrophage/monocyte cells (*TYROBP*, *LYZ*, *CD4*, and *AIF1*)^30^ (Supplementary Fig. 4C).

We recomputed the gene embedding and metagenes on only those cells classified as cancer by GeneVector with both adnexa and bowel samples. We found these cells exhibited large patient specific batch effects (Fig. 5C) and applied GeneVector batch correction (Fig. 5D, E). To understand the changes between primary and metastatic sites, we computed an average vector for all cells from the adnexa (**V**_adnexa_) as the primary site and bowel (**V**_bowel_) as the site of metastasis. We mapped the top 30 most similar metagenes to vectors representing expression gain in metastasis (**V**_adnexa_ - **V**_bowel_) (Fig. 5F) and expression loss (v_bowel_ - v_adnexa_) (Fig. 5G). Gene Set Enrichment Analysis (GSEA) using GSEAPY with Hallmark gene set annotations from Enrichr^35^ was performed to assess whether metagenes were enriched for genes from known pathways. Metagenes enriched for E2F targets and Epithelial-to-Mesenchymal Transition (EMT) pathway genes were found gained in metastasis. Conversely, the set of metagenes representative of loss from adnexa to bowel included MHC Class I (*HLA-A*, *HLA-B*, *HLA-C*, *HLA-E*, and *HLA-F*) and the transcriptional regulator *B2M*, suggesting a means of immune escape via loss of MHC Class I expression and higher immune pressure in metastatic sites may increase the potential fitness benefit of MHC Class I loss. For both the EMT and MHCI metagenes, pseudo-probabilities computed using GeneVector highlight pathway activity in either site in the UMAP embedding (Fig. 5H**)**. Computing the gene module scoring on the normalized, log-transformed expression^36^, we examined the change between sites in each patient for the EMT and MHCI metagenes. We found that the MHCI metagene is significantly downregulated in metastatic sites in four of six patients (Fig. 5I). Conversely, the EMT metagene was significantly up regulated in metastatic sites for three of six patients (Fig. 5J). The ability to phrase questions about transcriptional change as vector arithmetic provides a powerful platform for more complex queries than can be performed with differential expression analysis alone.

It is possible to perform latent space arithmetic operations on any embedding that is computed from a linear transformation of the gene space, including PCA. To assess the performance of latent space arithmetic using GeneVector and the principal components of a PCA decomposition of the gene expression matrix, we performed the same analysis on the cell-by-gene matrix. After clustering the gene embedding using the Leiden algorithm and we generated a list of candidate metagenes. We recomputed a representative vector for changes from bowel to adnexa by subtracting **V**_adnexa_ - **V**_bowel_ and selected the most similar metagenes. Like the GeneVector analysis, we recovered a metagene related to MHC Class I using the PCA embedding. However, several of the genes within this metagene were not present in the Reactome MHC Class I pathway (HSA-983169), in contrast to the GeneVector results which found a metagene containing only *HLA-A*, *HLA-B*, *HLA-C*, and *B2M*. To test if the unannotated genes defined by the PCA embedding were members of any gene signatures which contained HLA genes, we calculated the percentage of Reactome pathways that include individual gene pairs and plotted this as a heatmap over all genes in the metagene **(**Supplementary Fig. 5A). The genes *TMEM59, SERINC2, FOLR1*, and *WFDC2* were not found as a pair within any Reactome pathway. We concluded that the PCA embedding identified an MHC Class I metagene that was less consistent with previously annotated pathways than GeneVector.

Given that the metagenes are computed using Leiden clustering, the resolution parameter affects the coarseness of the PCA embedding. We performed a parameter sweep over resolution values and found that the genes identified by the PCA analysis are robustly clustered together over a wide range of resolutions (Supplementary Fig. 5B). In comparison, GeneVector identifies a metagene containing *HLA-A*, *HLA-B*, *HLA-C*, and *B2M* over a wide range of resolution parameters (Supplementary Fig. 5C). The interval of values used for the parameter sweep was selected to keep metagene membership between three and 50 genes and represents the most reasonable range of values for generating metagenes of this size. To understand why non MHCI-annotated genes appear in the context of MHC genes in the PCA embedding, we looked at differentially expressed genes up-regulated in adnexa over bowel. We found that while these genes do not appear in Reactome pathways together, they do appear significantly differentially expressed between adnexa and bowel. Here, we draw the conclusion that PCA combines multiple pathways to explain as much variance as possible when constructing successive orthogonal PCA components. As a result, PCA will combine multiple underlying sources of variance such as bowel/adnexa variation, and MHC I variation between cells. By contrast, GeneVector has no orthogonality constraints and is not constructed to maximize variance explained by individual vectors, allowing GeneVector to decompose pathway specific metagenes in a more flexible manner.

### Metagenes associated with cisplatin treatment resistance

**Fig. 6:**
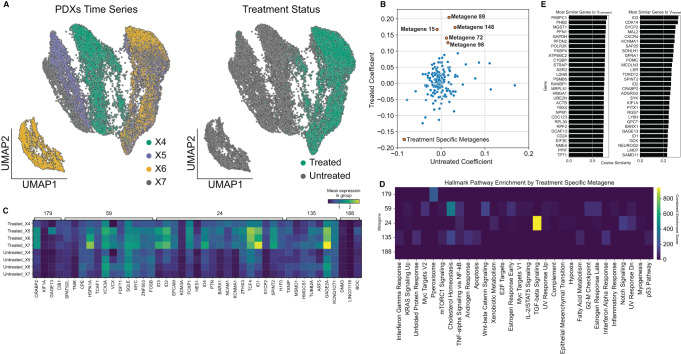
Analysis of Metagenes in Cisplatin-treated PDX Time Series. **A** UMAP of cisplatin treated PDX (patient derived xenograft) cells annotated by time point (left) and treatment status (right). **B** Metagenes plotted with respect to regression coefficients (β_treated_ and β_untreated_) over four timepoints in either treated or untreated cells with Bonferroni adjusted *p*-values = 0.001. **C** Hallmark combined enrichment scores for candidate chemo resistant metagenes. **D** Gene expression profiles for each timepoint for the five metagenes associated with increase in treatment. Metagene 24 is associated with TGF-beta signaling includes *EPCAM*, *FOXP1*, and *ID* family genes with known cisplatin resistance function. **E** Most similar genes for treated and untreated cells computed from cosine similarity to global vectors. Source data provided as a Source Data file.

Understanding the transcriptional processes that generate resistance to chemotherapies has immense clinical value. However, the transcriptional organization of resistance is complex with many parallel mechanisms contributing to cancer cell survival^37^. We analyzed longitudinal single cell RNA-seq collected from a triple negative breast cancer patient-derived xenograft model (SA609 PDX) along a treated and untreated time series^22^. Using a total of 19,799 cancer cells with treatment and timepoint labels (Fig. 6A), GeneVector was trained using unnormalized read counts to generate metagenes that identify programs potentially related to cisplatin resistance. For each metagene, we computed gene module scores over normalized and log-transformed expression^36^ over the four timepoint (X1, X2, X3, and X4) within the treated and untreated cells. Using these scores, we calculated linear regression coefficients over the four time points and selected candidate chemotherapy resistant and untreated metagenes (*β*_*treated*_ and *β*_*untreated*_*)* over a coefficient threshold (*β*_*treated*_ > 0.1 and Bonferroni adjusted *p* = 0.001) with an untreated coefficient less than the treated coefficient (*β*_*treated*_ *> β*_*untreated*_*)* **(**Fig. 6B). Mean log-normalized expression per gene was computed for each timepoint in five metagenes that were identified as treatment specific (Fig. 6C).

Pathway enrichment on each of the five metagenes using GSEAPY with Hallmark gene set annotations from Enrichr^35^ showed that metagene 24 was enriched for TGF-beta signaling (Fig. 6D), a pathway frequently up-regulated during chemo-resistance^38^. This metagene includes genes *ID1*, *ID2*, *ID3*, *ID4*, *EPCAM*, and *FOXP1*, all of which have been found to be overexpressed in chemotherapy-resistant samples in several cancer types^39–41^. GeneVector also identified these genes in global expression differences between treated and untreated cells from the set of most similar genes to vectors **V**_treated_ and **V**_untreated_ (Fig. 6E). Several studies have implicated multiple resistance mechanisms involving FOXP1 including transcriptional regulation, immune response, and MAPK signaling^42–44^. Additionally, GeneVector identifies *EPCAM*, whose high expression has been associated with increased viability of cancer cells in diverse cancer types^45^. *EPCAM* has been shown to have a role in resistance to chemotherapy in both breast and ovarian cancers through WNT signaling and Epithelial-Mesenchymal Transition (EMT)^46,47^.

## Discussion

In this paper we propose GeneVector, a method for building a latent vector representation of scRNA expression capturing the relevant statistical dependencies between genes. By borrowing expression signal across genes, GeneVector overcomes sparsity and produces an information dense representation of each gene. The resulting vectors can be used to generate a gene co-expression graph, and can be clustered to predict transcriptional programs, or metagenes, in an unsupervised fashion. Metagenes can be related to a cell embedding to identify transcriptional changes related to conditional labels or time points. We show that gene vectors can be used to annotate cells with a pseudo-probability, and that these labels are accurate with respect to previously defined cell types.

We show that a single GeneVector embedding can be used for many important downstream analyses. In interferon-stimulated PBMCs, we identify a cell type independent ISG metagene that summarizes interferon-stimulation across cell types and is not conflated with cell type signature. We demonstrate accurate cell type assignment across 18 different cancers in 181 patients described in the Tumor Immune Cell Atlas (TICA). In high grade serous ovarian cancer, we identify metagenes that describe transcriptional changes from primary to metastatic sites. Our results implicate the loss of MHC class I gene expression as a potential immune escape mechanism in ovarian cancer metastasis. In cisplatin treated TNBC PDXs, GeneVector uncovers transcriptional signatures active in drug resistance, most notably metagenes enriched in TGF-beta signaling. This signaling pathway is a cornerstone in cancer progression since it promotes EMT transition and invasion in advanced cancers; it is the target of various therapies, but success has been mixed^48^ making it even more important to employ tools that identify the multitude of players contributing to therapy response.

GeneVector can produce an interpretable batch correction by decomposing the derived correction vectors into transcriptional signatures. Using both the full PBMC dataset and simulated mixtures of CD14+ monocytes and CD4 T cells, we systematically evaluated the quality of batch correction using a series of benchmarking metrics (ARI, kBET, cLISI, and silhouette score)^49,50^ (Supplementary Fig. 6A–I). For the interferon beta stimulated PBMC dataset, we expect the proper batch correction to include ISGs, and indeed the batch correction vector had high cosine similarity to an ISG metagene. In simulated mixtures of cell types, we found that the similarity of the batch correction vector to ISGs was highest when the ratio of cell types was balanced in the unstimulated and stimulated batches, we obtained the best batch correction performance for balanced batches (Supplementary Fig. 6J). By contrast, when cell types were imbalanced, batch correction vectors had high similarity to cell type specific vectors (Supplementary Fig. 6K, L). Batch correction is challenging in the presence of batch-specific differences in cell type composition. As opposed to other batch correction methods, the GeneVector implementation is highly interpretable, so users can verify whether batch correction vectors are similar to cell type signatures or other biologically relevant metagenes.

Identifying correlations across scRNA data is a fundamental analysis task, necessary for identifying cells with similar phenotype or activity, or genes with similar pathways or functional relationships. As has been shown by us and in previous work^15,16,51^, the sparsity and non-linearity of scRNA data impact the performance of both standard measures of correlations between variables and global analysis of assumed linear correlations using PCA. While some methods tailor complex custom probabilistic models to the specific properties of scRNA data, GeneVector instead builds upon MI, a simple yet powerful tool for calculating the amount of information shared between two variables. We show that MI and the vector space trained from the MI matrix both capture relevant gene pair relationships including between TF activators and repressors and their targets. Nevertheless, GeneVector is unable to discern repression from activation, as it builds MI that is agnostic to the direction of the statistical dependency. Pearson correlation, theoretically sensitive to the direction of a dependency, also performs poorly. Due to the high-level of sparsity, absence of expression is not a significant event and repressed expression could just as easily be explained by under-sampling an expressed gene. We suggest that identifying negative regulation and mutually exclusive expression is one of the more difficult problems in scRNA-seq analysis.

As shown with correlation, the objective function employed in training GeneVector has a significant effect on the resulting gene embedding. Mutual information calculated empirically from the histogram of binned expression counts for gene pairs is limited by the available number of cells, fidelity of the counts, and discretization strategy. By modeling the underlying distribution for each gene more accurately, the joint probability distribution between genes can more accurately reflect expression-based relationships and improve model results. Additionally, while only a one-time cost, the MI calculation is computationally expensive. By improving the calculation of mutual information, others have achieved improved performance in related tasks including the identification of GRNs^52^.

The high dimensionality of scRNA and the vast complexity of biological systems to which it is applied necessitate analytical tools that facilitate intuitive and efficient data exploration and produce easily interpretable results. GeneVector performs upfront computation of a meaningful low dimensional representation, transforming sparse and correlated expression measurements into a concise vector space summarizing the underlying structure in the data. The resulting vector space is amenable to intuitive vector arithmetic operations that can be composed into higher level analyses including cell type classification, treatment related gene signature discovery, and identification of functionally related genes. Importantly, the vector arithmetic operations and higher-level analyses can be performed interactively, allowing for faster iteration in developing cell type and context specific gene signatures or testing hypotheses related to experimental covariates. GeneVector is implemented as a python package available on GitHub (https://github.com/nceglia/genevector) and installable via PIP.

## Methods

### Dataset preprocessing

Each single cell RNA-seq dataset in this study was processed using the Scanpy python library^7^. All highly variable gene selection was performed using the Seurat V3^6^ method implemented in Scanpy. We performed cell type classification on 23,764 cells from the Tumor Immune Cell Atlas (TICA)^20^ using raw read counts from 2,000 highly variable after removal of non-protein coding genes. We performed analysis of 6,855 cells composed of an interferon beta stimulated sample and a control sample using raw counts and cell type annotations obtained from the SeuratData R package^33^ and subset to 1000 highly variable genes. The fitness PDX dataset^22^ consisted of raw counts from 19,799 cancer cells with treatment and timepoint labels subset to 2000 highly variable genes. Coefficients and *p*-values were computed over gene module scores using the statsmodels python package. Cell type classification and vector arithmetic for the changes between metastatic and primary sites was performed on 270,833 cells from adnexa (primary) and bowel (metastasis) samples using 2000 highly variable genes. Normalization comparisons were made using the Scanpy normalize_total and log1p functions. All subsampling of datasets for simulated batch effects was performed using the Scanpy subsample function.

### Gene expression mutual information

In NLP applications, vector space models are trained by defining an association between words that appear in the same context. In single cell RNA sequencing data, we can redefine this textual context as co-expression within a given cell and mutual information across cells. The simplest metric to define association is the overall number of co-expression events between genes. However, the expression profiles over cells may differ due to both technical and biological factors. To summarize the variability in this relationship, we generate a joint probability distribution on the co-occurrence of read counts. The ranges of each bin are defined separately for each gene based on a user defined number of quantiles. By defining the bin ranges separately, the lowest counts in one gene can be compared directly to the lowest counts in another gene without need for further normalization. Using the joint probability distribution, we compute the mutual information between genes defined in Eq. 1. The mutual information value is subsequently used as the target in training the model, allowing us to highlight the relationship between genes as a single-valued quantity.

1 $$I\left({{{{{{\bf{G}}}}}}}_{{{{{{\bf{i}}}}}}},{{{{{{\bf{G}}}}}}}_{{{{{{\bf{j}}}}}}}\right)=\mathop{\sum }\limits_{i}^{n}\mathop{\sum }\limits_{j}^{n}p\left({{{{{{\bf{G}}}}}}}_{{{{{{\bf{i}}}}}}},{{{{{{\bf{G}}}}}}}_{{{{{{\bf{j}}}}}}}\right)\log \left(\frac{p\big({{{{{{\bf{G}}}}}}}_{{{{{{\bf{i}}}}}}},{{{{{{\bf{G}}}}}}}_{{{{{{\bf{j}}}}}}}\big)}{p\left({{{{{{\bf{G}}}}}}}_{{{{{{\bf{i}}}}}}}\right),p\big({{{{{{\bf{G}}}}}}}_{{{{{{\bf{j}}}}}}}\big)}\right)$$

Equation 1: Mutual information between **G**_i_ and **G**_j_ computed on the empirical joint probability distribution over expression bins for **G**_i_ and **G**_j_.

### Model training

A neural network is constructed from a single hidden layer corresponding to the size of the latent space vectors. A set of independently updated weights connects the one-hot encoded input and output layers that are defined from each pair of genes. These weights, **w**_1_ and **w**_2_, are matrices with dimensions equal to *N* expressed genes by *I* hidden units. Initial values for **w**_1_ and **w**_2_ are generated uniformly on the interval −1 to 1. The objective function, as a minimization of least squares, is defined in Eq. 2. The final latent space, defined as the gene embedding, is computed as the vector average of weights *w*_*1*_ and *w*_*2*_. For NLP applications, this is a preferred approach over selecting either weight matrix^4^. Each co-expressed gene pair is used as a single training example. The maximum number of examples for a full training epoch is given by the total number of co-expressed gene pairs. Weights are batch updated with adaptive gradient descent (ADADELTA)^53^. Training is halted at either a maximum number of epochs, or when the change in loss falls below a specified threshold. The results presented used unnormalized integer read counts, but any matrix can be used as input to the model. The model is implemented in PyTorch.

2 $$J={\left({{{{{{{\bf{w}}}}}}}_{i}}^{T}{\hat{{{{{{\bf{w}}}}}}}}_{j}-C*I({{{{{{\bf{G}}}}}}}_{{{{{{\bf{i}}}}}}},{{{{{{\bf{G}}}}}}}_{{{{{{\bf{j}}}}}}})\right)}^{2}$$

Equation 2: Objective function for weights corresponding to gene **G**_**i**_ and **G**_**j**_, where $I({{{{{{\bf{G}}}}}}}_{{{{{{\bf{i}}}}}}},{{{{{{\bf{G}}}}}}}_{{{{{{\bf{j}}}}}}})$ is the mutual information and *C* is a constant.

### Gene vectors

The cosine similarity function, defined in Eq. 3, is used to measure similarity between vectors, defined as 1 - cosine distance or dot product. Values closer to 1 indicate strong association within the dataset. A gene vector is defined as the learned weights in the gene embedding for a particular gene. Vectors describing groups of cells are generated by computing a weighted average vector, described in Eq. 4, from a set of individual gene vectors.

3 $${cosinesimilarity}=1-\frac{{{{{{\bf{A}}}}}}*{{{{{\bf{B}}}}}}}{\left|\left|{{{{{\bf{A}}}}}}\right|\right |*\left|\left|{{{{{\bf{B}}}}}}\right|\right|}$$

Equation 3: Cosine similarity as the dot-product between feature vectors $A$ and $B$.

To assign a vector to each cell, the average vector is computed across the gene embedding weighted by the normalized, log-transformed expression observed in each cell. The matrix of all cell vectors in the dataset is defined as the cell embedding. This cell embedding can be used in place of PCA or embeddings obtained with variational auto-encoders. Each cell vector maintains a linear relationship with the gene embedding. The computation of vectors describing groups of cells is described in Eq. 4.

4 $${{{{{{\bf{C}}}}}}}_{{{{{{\bf{k}}}}}}}=\frac{{\sum }_{i=1}^{n}{{{{{\bf{w}}}}}}_{i\ast}{\underline{{{{{{\bf{x}}}}}}}_{{{{{{\bf{i}}}}}}}}}{{\sum }_{i=1}^{n}{{{{{\bf{w}}}}}}_{i}}$$

Equation 4: Vector ${C}_{k}$ defined as the $k$ th component of the cell embedding computed from the average mean of vectors ${\underline{x}}_{i\to n}$ where $n$ is equal to the number of hidden units.

### Co-expression similarity graph

A co-expression similarity graph is constructed from cosine similarity between each pair of genes using Scanpy neighborhood function with a default value for *k* of 15. A node in the graph represents a single gene and edges are weighted by cosine similarity. To generate metagenes, we apply Leiden clustering to the neighborhood graph with a user defined resolution argument. We have found that metagene clustering is robust to a range of resolution values ranging from 15 to 1000 when assessing the identification of an MHCI-like metagene in a single patient in the SPECTRUM cohort (Supplementary Fig. 5C).

### Cell type assignment

A set of cell types with user defined marker genes are used to perform a pseudo-probabilistic phenotype assignment in a single cell. A representative vector for the cell type is computed from the user defined marker genes weighted by the normalized and log-transformed expression of the cell to be classified. The cosine distances of each cell type vector with cell vector are passed through a SoftMax function, given in Eq. 5, to provide a pseudo-probability distribution for each phenotype. The argument maximum of this distribution is used to classify the most likely cell type for a given cell. This procedure is repeated for every cell in the dataset.

5 $$\sigma (z)_{i}=\frac{{e}^{{Z}_{i}}}{{\sum }_{j=1}^{n}{e}^{{Z}_{i}}}$$

Equation 5: SoftMax function where $z$ is the set of cosine similarities for $n$ cell types.

### Generation of predictive genes

The method of phenotype assignment can be reversed to produce a set of genes that are most similar, or predictive, to any group of cells. The cell vectors belonging to this group can be averaged to generate a group vector representing a label in the dataset. The transcriptional signature associated with this group of cells can be computed using vector arithmetic **V**_group_ – **V**_dataset_, where **V**_dataset_ is the average vector over all cells. Gene vectors are sorted by cosine similarity to the group vector to produce a ranked list of candidate genes that are most like the set of cells in the group.

### Batch correction

Batch correction is applied to cells over a given set of batch labels with the goal of correcting the difference between these batches and a user-specified reference batch. With the set of cells labeled for a batch, we can compute an average batch vector. A correction vector can then be computed for each batch using vector arithmetic to describe the direction and magnitude of the change from the batch to the reference. The correction vector is then added to each cell in the batch. After computing the cell embedding, the following procedure is applied to each batch label:

1. Compute the average vector **V**_batch_ for a set of cells.
2. Compute the reference vector **V**_reference_ for cells in the reference batch.
3. Compute a correction vector **V**_correction_ = **V**_reference_ – **V**_batch_.
4. For each cell in the batch, subtract **V**_correction_ from each cell vector.
5. Repeat for all *n* batches.

### Co-expressed and mutually exclusive markers

Cell type marker gene pairs used to benchmark normalization methods by identifying common differentially expressed genes in T cell, B cell, and monocyte lineage cell types across multiple datasets and extracting the top 10 markers of each cell type in each dataset. The union of extracted marker genes over all cell types for a given lineage (T cell, B cell, or monocyte) were ranked by the number of datasets where that gene was a marker. All combinations of markers in the top 10 ranked genes that were unique to a cell type were selected as the co-expressed pairs for T cell, B cell, and monocyte lineages. Mutually exclusive markers were defined as all combinations of gene pairs that were not found together in a single lineage.

### Time-series analysis of metagenes

Linear regression is applied to gene module scores computed over normalized, log-transformed expression^36^ over a series of time points for each metagene resulting in a *p*-value and slope coefficient from each series. Metagenes with a Bonferroni adjusted *p* = 0.001 and a treated coefficient greater than the untreated coefficient, with a minimum threshold of 0.1 were selected as treatment specific gene programs.

### Reporting summary

Further information on research design is available in the Nature Portfolio Reporting Summary linked to this article.

## Supplementary information

Supplementary Information

Reporting Summary

## Data Availability

No novel sequencing data were generated for this current study. All pre-processed single cell RNA-seq data analyzed in this study are available in the Zenodo database here [10.5281/zenodo.8079610]^54^. The Tumor Immune Cell Atlas (TICA) processed data can be accessed here [https://zenodo.org/record/4263972] and the raw data has been deposited in the Gene Expression Ominibus under the accession GSE158803. The PBMC dataset can be accessed through SeuratData R package^32,33^ and has been deposited in the Gene Expression Omnibus under the accession number GSE96583. The SPECTRUM High Grade Serous Ovarian Cancer dataset can be accessed here [https://cellxgene.cziscience.com/collections/4796c91c-9d8f-4692-be43-347b1727f9d8]. Raw 10x sequencing for the Fitness dataset is available from the European Genome-Phenome under study ID EGAS00001004448. Source data are provided with this paper.

## Source data

Source Data

## Code availability

The authors declare that the code needed to reproduce analysis and findings of this study are available in the GitHub repository (https://github.com/nceglia/genevector) and the GeneVector python package can be installed directly from using the Python installer package (PIP). Code released with this manuscript can be found at 10.5281/zenodo.8079885^55^.
