# Supplementary material for: Identification of transcriptional programs using dense vector representations defined by mutual information with GeneVector
Source: Nat Commun. 2023 Jul 20;14:4400. doi: 10.1038/s41467-023-39985-2 (PMC10359421; doi:10.1038/s41467-023-39985-2)
Supplement: Supplementary file 1 — Supplementary Information [file 41467_2023_39985_MOESM1_ESM.pdf]

Supplementary Information

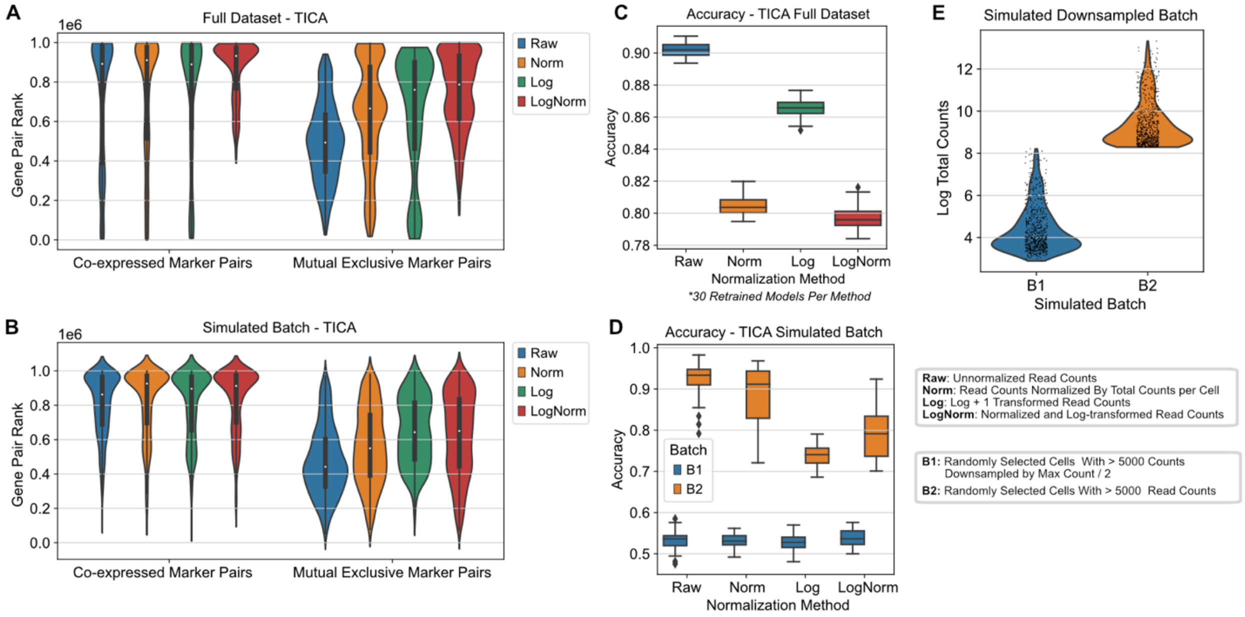

Supplemental Figure 1 - GeneVector Similarity and Classification Performance with Simulated Batch Effects: A-B) Genes pairs were ranked by cosine similarity from embeddings generated from multiple training runs on the both the TICA dataset and subset datasets with artificially generated batch effect using four different normalization procedures (raw counts, normalized counts, log transformed counts, and normalized and log transformed counts). The distribution of ranks is shown for both co-expressed marker pairs in the full dataset (n=33984) and simulated dataset (n=14484) compared to mutually exclusive cell type marker pairs in the full dataset (n=224350) and simulated dataset (n=13632) (Methods: Co-expressed and Mutually Exclusive Markers). The center of the violin is denoted by the median, a single white circle. The thicker vertical bar of each violin is defined by the lower quartile (25th percentile) and the upper quartile (75th percentile). The thinner bar extends from the IQR range and represent the data points that fall within 1.5 times the interquartile range (IQR) from the lower and upper quartiles. The width of the violin represents kernel density estimation at the horizontal value. C-D) Accuracy using cell type markers for coarse cell types (T Cell, B/Plasma, and Myeloid) (Methods: Cell Type Assignment) are shown for both the TICA dataset (n=499) and subset datasets (n=158) with artificially generated batch effect for each normalization method. The center of the box plot is denoted by the median, a horizontal line dividing the box into two equal halves. The bounds of the box are defined by the lower quartile (25th percentile) and the upper quartile (75th percentile). The whiskers extend from the box and represent the data points that fall within 1.5 times the interquartile range (IQR) from the lower and upper quartiles. Any data point outside this range is considered an outlier and plotted separately. E) Log-transformed total counts from batches in subset datasets with artificially generated batch effect. Two simulated batches were generated by randomly selecting half the cells (batch B1) in the TICA dataset with greater than 5000 counts. Batch 1 (B1) counts were down sampled to half the original total counts and batch 2 (B2) was unaltered.

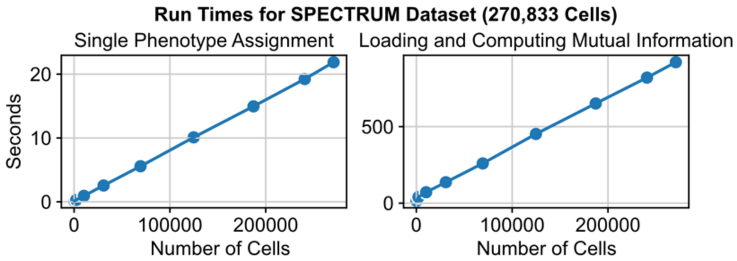

Supplemental Figure 2: Run times for increasing number of cells for assigning a phenotype to each cell using five gene markers and the time needed to compute the mutual information for 1,000 genes. Both operations scale linearly with input size. Each time was computed on a subsample of the SPECTRUM dataset.

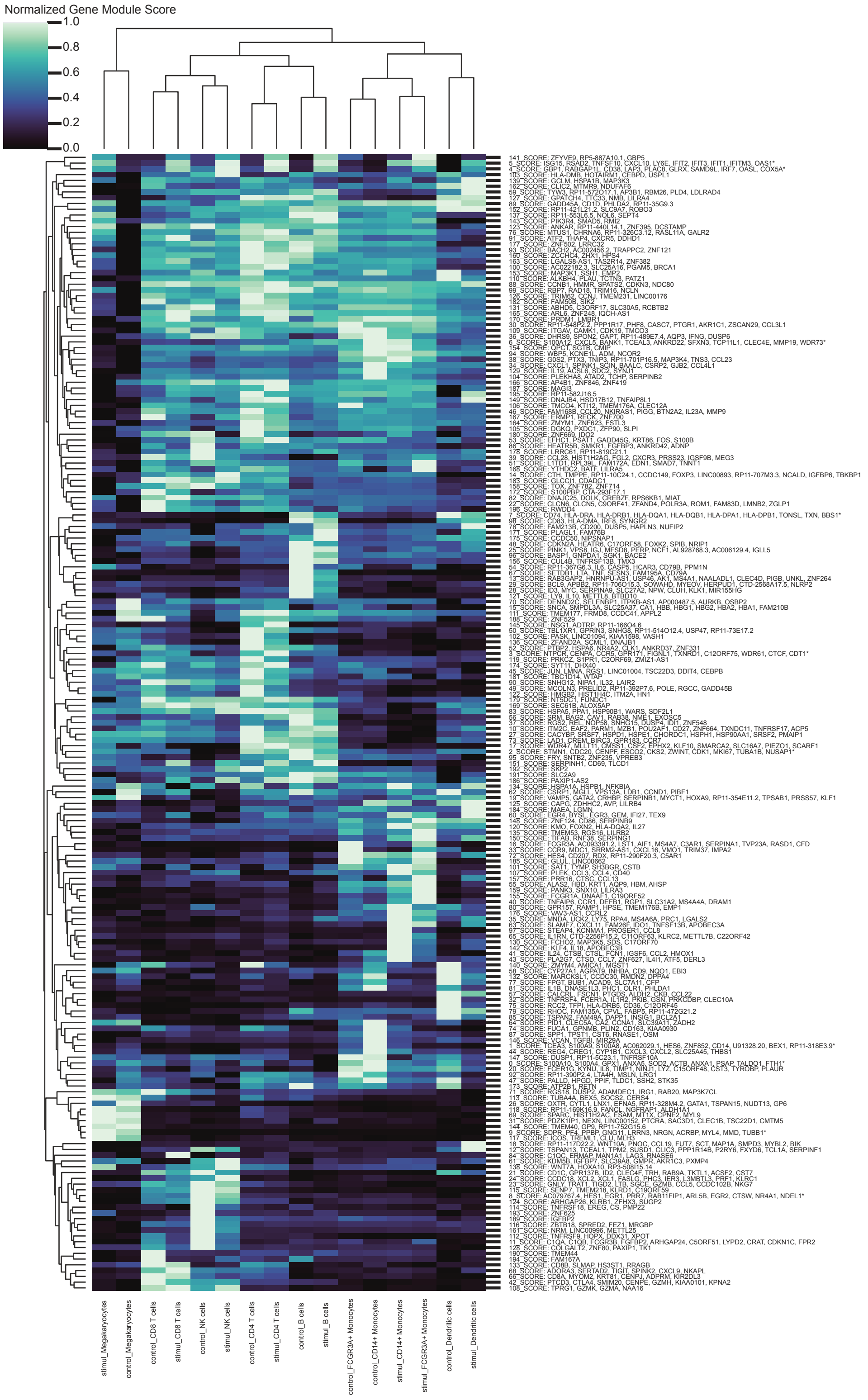

Supplemental Figure 3: Gene module scores for all metagenes identified in interferon stimulated PBMCs. *****Indicates additional gene membership. Metagenes programs can be found for each individual cell type and treatment condition including very small cell populations like Megakaryocytes (132 of 14,038 cells). Data available in Source Data.

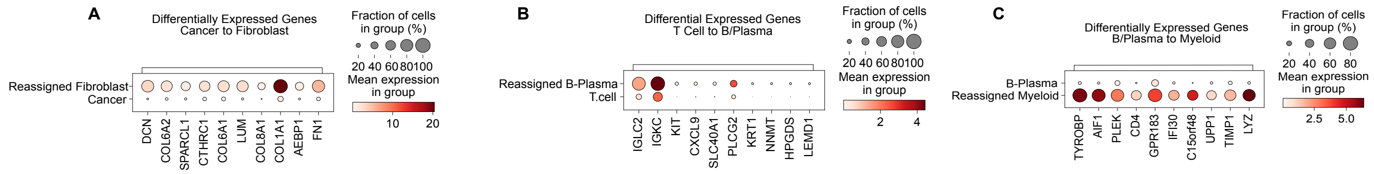

Supplemental Figure 4 – Differentially Expressed Genes for Reclassified Cells in SPECTRUM Dataset: A) Differentially expressed genes in cells classified as fibroblast cells that were classified as cancer using CellAssign highlights the *COL1A1*. B) Differentially expressed genes in cells classified as B/Plasma cells that were classified as T cells using CellAssign highlights the genes *IGKC* and *IGLC2*. C) Differentially expressed genes in cells classified as Myeloid cells that were classified as B/Plasma using CellAssign highlights the genes *TYROBP*, *AIF1*, *CD4*, and *LYZ*.

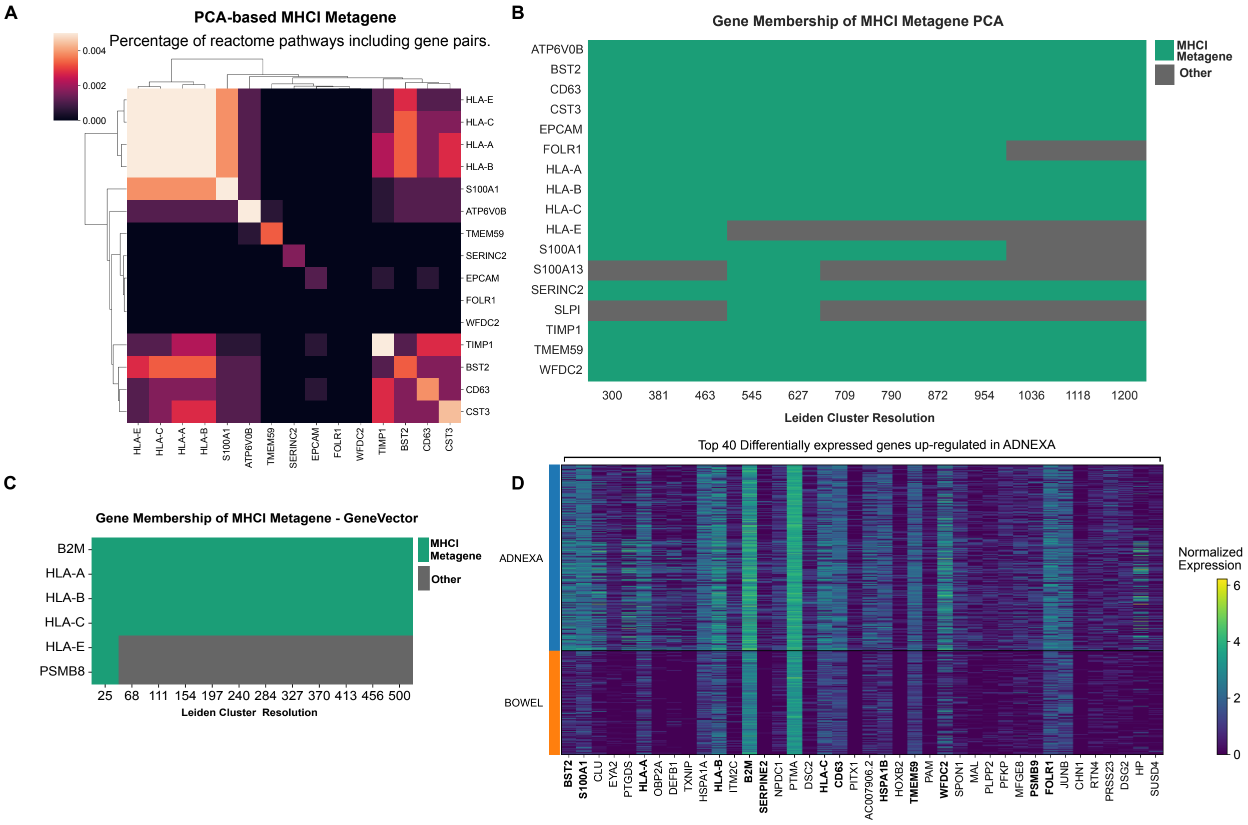

Supplemental Figure 5 – Comparison of Gene Membership in Metagenes Identified by GeneVector with PCA Loadings: A) Percentage of Reactome pathways where gene pairs from the PCA-based MHCI metagene were found together. B) Gene membership in MHCI metagene over multiple Leiden resolution values highlights robust clustering of HLA genes and non-pathway specific genes. C) Gene membership of MHCI metagene generated from GeneVector robustly selects HLA specific genes. D) Differentially expressed genes up regulated in adnexa includes non-specific pathway genes alongside MHCI genes. Data available as Source Data.

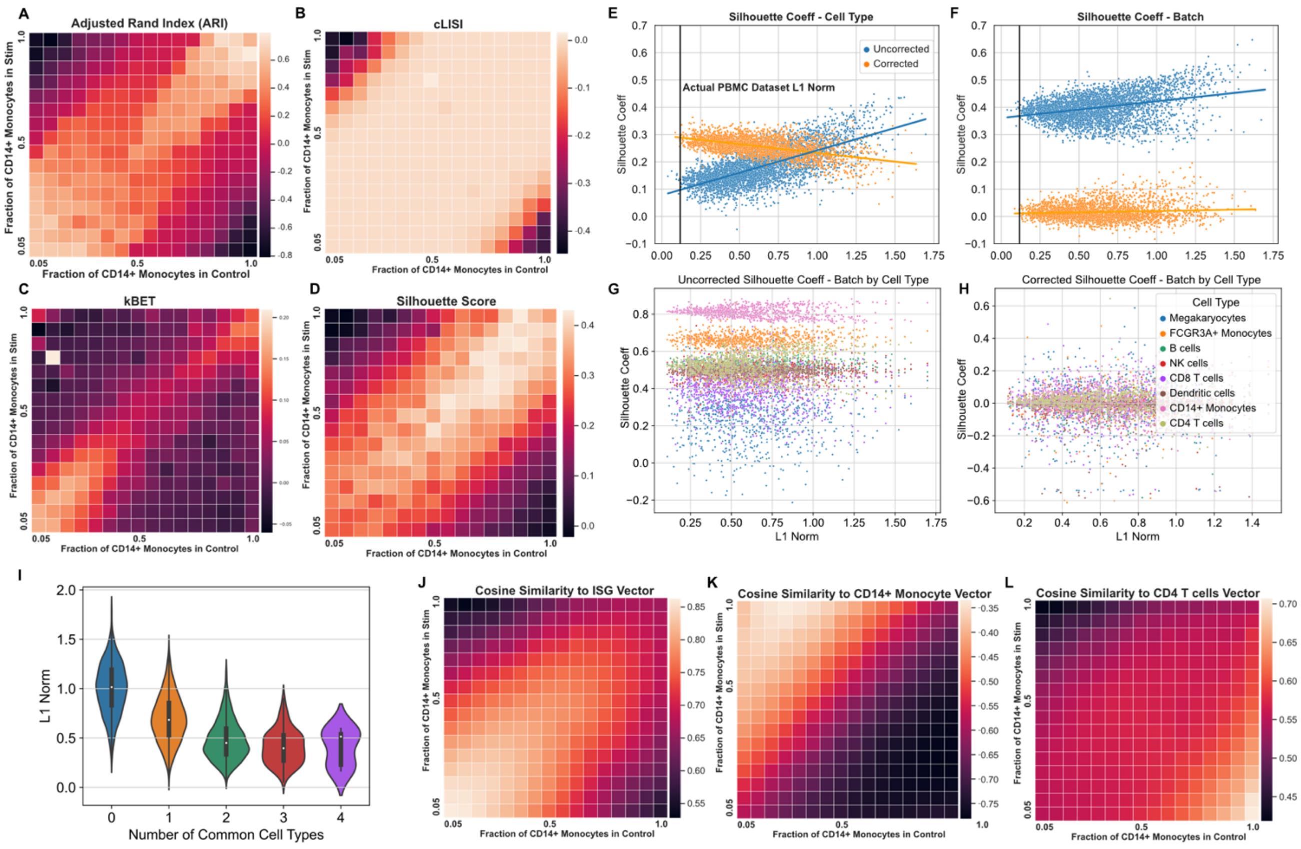

Supplemental Figure 6 – Benchmarking Metrics for GeneVector Batch Correction in PBMCs: A-D) Benchmarking metrics on a subset of the PBMC dataset with varying ratios of CD14+ monocytes and CD4 T cells includes Adjusted Rand Index (ARI), cLISI, kBET, and silhouette score. E-F) Silhouette coefficient score over cell type and batch for different L1 distances of cell type proportion between stimulated and control batches. G-H) Silhouette score over batch label computed within each cell type for uncorrected and corrected embeddings. I) Distribution of L1 norm distances over the number of conserved cell types between batches after random down sampling (n=4559). The center of the violin is denoted by the median, a single white circle. The thicker vertical bar of each violin is defined by the lower quartile (25th percentile) and the upper quartile (75th percentile). The thinner bar extends from the IQR range and represent the data points that fall within 1.5 times the interquartile range (IQR) from the lower and upper quartiles. The width of the violin represents kernel density estimation at the horizontal value. J-L) Cosine similarity of batch correction vector to ISG vector, CD14 monocyte vector, and CD4 T cell vector for varying ratios of CD14+ monocytes and CD4 T cells. Data available as Source Data.
