# Supplementary material for: Identification of transcriptional programs using dense vector representations defined by mutual information with GeneVector
Source: Nat Commun. 2023 Jul 20;14:4400. doi: 10.1038/s41467-023-39985-2 (PMC10359421; doi:10.1038/s41467-023-39985-2)
Supplement: Supplementary file 2 — Reporting Summary [file 41467_2023_39985_MOESM2_ESM.pdf]

nature portfolio

Corresponding author(s): Nicholas Ceglia

Last updated by author(s): Jun 25, 2023

Reporting Summary

Nature Portfolio wishes to improve the reproducibility of the work that we publish. This form provides structure for consistency and transparency in reporting. For further information on Nature Portfolio policies, see our Editorial Policies and the Editorial Policy Checklist.

Statistics

For all statistical analyses, confirm that the following items are present in the figure legend, table legend, main text, or Methods section.

n/a	Confirmed	
☐	☒	The exact sample size (n) for each experimental group/condition, given as a discrete number and unit of measurement
☒	☐	A statement on whether measurements were taken from distinct samples or whether the same sample was measured repeatedly
☐	☒	The statistical test(s) used AND whether they are one- or two-sided<br>*Only common tests should be described solely by name; describe more complex techniques in the Methods section.*
☐	☒	A description of all covariates tested
☒	☐	A description of any assumptions or corrections, such as tests of normality and adjustment for multiple comparisons
☐	☒	A full description of the statistical parameters including central tendency (e.g. means) or other basic estimates (e.g. regression coefficient) AND variation (e.g. standard deviation) or associated estimates of uncertainty (e.g. confidence intervals)
☐	☒	For null hypothesis testing, the test statistic (e.g. F, t, r) with confidence intervals, effect sizes, degrees of freedom and P value noted<br>*Give P values as exact values whenever suitable.*
☒	☐	For Bayesian analysis, information on the choice of priors and Markov chain Monte Carlo settings
☒	☐	For hierarchical and complex designs, identification of the appropriate level for tests and full reporting of outcomes
☒	☐	Estimates of effect sizes (e.g. Cohen's d, Pearson's r), indicating how they were calculated

Our web collection on statistics for biologists contains articles on many of the points above.

Software and code

Policy information about availability of computer code

Data collection	10k PBMC processed dataset are available in the code package: https://github.com/nceglia/genevector/blob/main/example/PBMC.h5ad.<br>TICA Cell Atlas Dataset is available at https://zenodo.org/record/4263972/files/TICAtlas_downsampled_1000.h5ad. Processed data can be provided immediately on request.<br>Raw SA609 Cell Line 10x scRNA-seq are available from the European Genome-Phenome under study ID EGAS00001004448. Processed data can be provided immediately on request.<br>SPECTRUM HGSOC 10x dataset will be made available on publication of this study. Processed data can be provided immediately on request for the single patient results generated for this manuscript.
Data analysis	Source code for GeneVector is available at: https://github.com/nceglia/genevector<br>Installation and basic functionality described in README: https://github.com/nceglia/genevector/blob/main/README.md<br>Jupyter notebooks for analysis: https://github.com/nceglia/genevector/tree/main/example<br>List of additional Python packages and versions for installation and running examples:

For manuscripts utilizing custom algorithms or software that are central to the research but not yet described in published literature, software must be made available to editors and reviewers. We strongly encourage code deposition in a community repository (e.g. GitHub). See the Nature Portfolio guidelines for submitting code & software for further information.

Data

Policy information about availability of data

All manuscripts must include a data availability statement. This statement should provide the following information, where applicable:

- Accession codes, unique identifiers, or web links for publicly available datasets
- A description of any restrictions on data availability
- For clinical datasets or third party data, please ensure that the statement adheres to our policy

The peripheral blood mononuclear cells (PBMCs) dataset is publicly available on the Yosef Lab website (https://github.com/YosefLab/scVI-data/raw/master/pbmc_10k_protein_v3.h5ad). The processed file containing only the highly variable genes is available in the GeneVector package. The down-sampled tumor immune cell atlas dataset is publicly available on Zenodo (https://zenodo.org/record/4263972/files/TICAtlas_downsampled_1000.h5ad). The original, unprocessed SA609 cell line dataset can be found at the European Genome-Phenome under study ID (EGAS00001004448). The SPECTRUM HGSOC dataset is not publicly available, but will be released in the GEO database after publication. Processed files for any dataset with highly variable gene selection can be provided immediately on request.

Human research participants

Policy information about studies involving human research participants and Sex and Gender in Research.

Reporting on sex and gender	No label defining sex or gender was reported or used to generate results.
Population characteristics	No human participants were collected in the generation of these results.
Recruitment	No recruitment for this study was performed.
Ethics oversight	No ethical oversight committee was involved in the construction of this software tool.

Note that full information on the approval of the study protocol must also be provided in the manuscript.

Field-specific reporting

Please select the one below that is the best fit for your research. If you are not sure, read the appropriate sections before making your selection.

☒ Life sciences ☐ Behavioural & social sciences ☐ Ecological, evolutionary & environmental sciences

For a reference copy of the document with all sections, see nature.com/documents/nr-reporting-summary-flat.pdf

Life sciences study design

All studies must disclose on these points even when the disclosure is negative.

Sample size	Sample size of each dataset is taken as the number of cells provided in each dataset.
Data exclusions	The number of genes used for generation of results was subset to the highly variable genes using the Seurat V3 method as implemented in Scanpy (https://scanpy.readthedocs.io/en/stable/generated/scanpy.pp.highly_variable_genes.html). The total number of genes used for each dataset is indicated in the results section.
Replication	No replicates were provided with each dataset.
Randomization	No randomization was necessary at any stage. The full set of cells (or subset based on condition) was used for each result generated from the accompanying dataset.
Blinding	No blinding was relevant to these results.

Reporting for specific materials, systems and methods

We require information from authors about some types of materials, experimental systems and methods used in many studies. Here, indicate whether each material, system or method listed is relevant to your study. If you are not sure if a list item applies to your research, read the appropriate section before selecting a response.

Materials & experimental systems

n/a	Involved in the study
☒	☐ Antibodies
☒	☐ Eukaryotic cell lines
☒	☐ Palaeontology and archaeology
☒	☐ Animals and other organisms
☒	☐ Clinical data
☒	☐ Dual use research of concern

Methods

n/a	Involved in the study
☒	☐ ChIP-seq
☒	☐ Flow cytometry
☒	☐ MRI-based neuroimaging
